# Supplementary material for: Multivariate phenotype analysis enables genome-wide inference of mammalian gene function
Source: PLoS Biol. 2022 Aug 9;20(8):e3001723. doi: 10.1371/journal.pbio.3001723 (PMC9391051; doi:10.1371/journal.pbio.3001723)
Supplement: S1 Note — This note details the technical aspects of the UV model introduced in Results–Univariate model. (PDF) [file pbio.3001723.s001.pdf]

Supplementary Note 1. Univariate model details.

A transformation was applied to each quantitative phenotype separately, and to data from across all phenotyping centres at once. For any quantitative phenotype with some observations ≤ 0, a constant was added to all observations prior to transformation in order to satisfy: $\min(y) = (\max(y) - \min(y))/100$. Phenotypes were then Box-Cox transformed with the exponent λ constrained to be in $\lambda \in \{-2, -1.5, \ldots, 1.5, 2\}$ and chosen to maximise the likelihood with respect to λ under an ordinary Gaussian linear model applied to data from baseline animals with sex and day as covariates. After Box-Cox transformation, data for each centre-phenotype pair were scaled to zero median and unit median absolute deviation, and then winsorized at ± 20 to bound the influence of extreme data points.

Transformed quantitative phenotype p for KO line g were analysed under a Gaussian-response Bayesian multilevel model with day ($\boldsymbol{\alpha}^{\text{day}}$), litter ($\boldsymbol{\alpha}^{\text{litter}}$), genotype ($\theta_{pg}^{\text{UV}}$), sex ($\boldsymbol{\beta}^{\text{sex}}$), strain ($\boldsymbol{\beta}^{\text{strain}}$), investigator ($\boldsymbol{\beta}^{\text{inv}}$) and metadata group ($\boldsymbol{\beta}^{\text{meta}}$) as covariates, and with a penalized spline to account for systematic temporal trends in baseline animal measurements. The penalized spline was fitted as described in chapter 16 of [1], with the pure cubic polynomial component having coefficients $\boldsymbol{\beta}^{\text{poly}}$, and the full cubic spline's basis functions having coefficients α_k^{spl} which were regularised via a hierarchical model with variance component σ_{spl}^2. Day and litter effects were modelled hierarchically with variance components σ_{day}^2 and σ_{litter}^2. The residual variance is denoted by σ_{resid}^2. For any particular mutant line the analysis was restricted to data from that line along with data from all baseline animals at the same centre. The model was:

$$
\begin{aligned}
y_i &\sim N\left(\mu_i,\ \sigma_{\text{resid}}^2\right) \\
\mu_i &= \theta_{pg}^{\text{UV}} \mathbb{I}(\text{animal } i \text{ is in line } g) + \alpha_{d[i]}^{\text{day}} + \alpha_{l[i]}^{\text{litter}} + \sum_{k=1}^{K+3} \alpha_k^{\text{spl}} f_k\left(t_{d[i]}\right) + \\
&\quad \beta_{s[i]}^{\text{sex}} + \beta_{s[i],g[i]}^{\text{sex,geno}} + \beta_{j[i]}^{\text{strain}} + \beta_{v[i]}^{\text{inv}} + \beta_{m[i]}^{\text{meta}} + \sum_{p=1}^{3} \beta_p^{\text{poly}} t_{d[i]}^p \\
\alpha_d^{\text{day}} \mid \sigma_{\text{day}}^2 &\sim N\left(0,\ \sigma_{\text{day}}^2\right),\ \text{for } d = 1, \ldots, D \\
\alpha_l^{\text{litter}} \mid \sigma_{\text{litter}}^2 &\sim N\left(0,\ \sigma_{\text{litter}}^2\right),\ \text{for } l = 1, \ldots, L \\
\alpha_k^{\text{spl}} \mid \sigma_{\text{spl}}^2 &\sim N\left(0,\ \sigma_{\text{spl}}^2\right),\ \text{for } k = 1, \ldots, K+3
\end{aligned}
$$

where g indexes genotype, s sex, j strain, v investigator, and m metadata group; t_d is the time point corresponding to the dth day. The model adjusts for potential sex-genotype interaction effects [2], with a sum-to-zero contrast constraint—i.e. $\sum_{s \in \{\text{M,F}\}} \beta_{s,g}^{\text{sex,geno}} \equiv 0$ for each g—meaning that the main genotype effect is interpretable as the mean of the male and female genotype effects. The functions $f_k(\cdot)$ denote basis functions of a B-spline basis for a cubic spline with knots at regularly spaced quantiles of the empirical distribution of days, and the number of knots, K, rounded down from the number of unique days divided by 10.

Non-informative priors were specified for β and σ^2 within the conjugate prior families available in the software package used (MCMCglmm [3, 4]). The location parameters β were allocated independent Normal(mean $= 0$, variance $= 100$) priors. The variance parameters σ^2 were allocated independent Inverse-gamma(shape $= 0.01$, rate $= 0.01$) priors.[1]

[1] A non-informative Inverse-gamma(ε, ε) prior with small ε is a common but pragmatic choice for variance components, and we were guided by what was available in the software package used. It is known that there can be a degree of posterior sensitivity to the particular choice of ε (e.g. as ε varies from 0.01 to 0.001) [5]. In future methods development we would prefer a non-informative half-Cauchy prior as suggested by [5].

References

[1] Ruppert, D., Wand, M. P. & Carroll, R. J. *Semiparametric Regression*. Cambridge Series in Statistical and Probabilistic Mathematics (Cambridge University Press, 2003), first edn.

[2] Karp, N. A. *et al.* Prevalence of sexual dimorphism in mammalian phenotypic traits. *Nature Communications* **8**, 15475 (2017). URL `http://www.nature.com/doifinder/10.1038/ncomms15475`.

[3] Hadfield, J. D. MCMC Methods for Multi-Response Generalized Linear Mixed Models: The MCMCglmm R Package. *Journal of Statistical Software* **33**, 1–22 (2010). URL `http://www.jstatsoft.org/v33/i02`.

[4] R Development Core Team. *R: A Language and Environment for Statistical Computing*. R Foundation for Statistical Computing, Vienna, Austria (2018). URL `http://www.r-project.org`.

[5] Gelman, A. Prior distributions for variance parameters in hierarchical models. *Bayesian Analysis* **1**, 515–533 (2006).
