# Supplementary material for: Multivariate phenotype analysis enables genome-wide inference of mammalian gene function
Source: PLoS Biol. 2022 Aug 9;20(8):e3001723. doi: 10.1371/journal.pbio.3001723 (PMC9391051; doi:10.1371/journal.pbio.3001723)
Supplement: S2 Note — This note contains technical details of the EM algorithm introduced in Methods–EM algorithm. (PDF) [file pbio.3001723.s002.pdf]

Supplementary Note 2. EM algorithm.

The multivariate model, building on the models introduced in [1, 2], can be expressed as:

$$p(\hat{\boldsymbol{\theta}}_{\cdot g}^{\mathrm{UV}} \mid \boldsymbol{R}) = \mathrm{N}(\hat{\boldsymbol{\theta}}_{\cdot g}^{\mathrm{UV}} \mid \boldsymbol{\theta}_{\cdot g},\ \hat{\boldsymbol{S}}_g^{\mathrm{UV}} \boldsymbol{R} \hat{\boldsymbol{S}}_g^{\mathrm{UV}}) \tag{1}$$

$$p(\boldsymbol{\theta}_{\cdot g} \mid \boldsymbol{\Sigma}_{1:S}, \boldsymbol{\pi}) = \sum_{m=1}^{M} \sum_{s=1}^{S} \pi_{ms} \mathrm{N}(\boldsymbol{\theta}_{\cdot g} \mid \boldsymbol{0},\ \omega_m \boldsymbol{\Sigma}_s)\ . \tag{2}$$

We constrain $\boldsymbol{\Sigma}_s$ to factor-model form (see e.g. [3]):

$$\boldsymbol{\Sigma}_{\boldsymbol{s}} = \boldsymbol{W}_s \boldsymbol{W}_s^T + \boldsymbol{\Psi}_s\ , \tag{3}$$

where $\boldsymbol{W}_s$ is a $P \times K$ matrix, and $\boldsymbol{\Psi}_s$ a diagonal $P \times P$ matrix having positive diagonal elements. Under an improper uniform prior on the factor-model space, our specified prior for $\boldsymbol{\Sigma}_{\boldsymbol{s}}$ is

$$p(\boldsymbol{\Sigma}_s) \propto \begin{cases} 1 & \boldsymbol{\Sigma}_s \in \mathcal{W}_K \\ 0 & \text{otherwise} \end{cases} \tag{4}$$

$$\mathcal{W}_K := \{\boldsymbol{W}\boldsymbol{W}^T + \boldsymbol{\Psi}_s : \boldsymbol{W} \in \mathbb{R}^{P \times K},\ \boldsymbol{\Psi}_s \text{ diagonal with } [\boldsymbol{\Psi}_s]_{jj} \geq 0\ \forall j\}\ . \tag{5}$$

The Dirichlet prior we specify for $\boldsymbol{\pi}$ is

$$\mathrm{vec}(\boldsymbol{\pi}) \sim \mathrm{Dirichlet}(\mathrm{vec}(\boldsymbol{\pi}) \mid \alpha_{11}, \ldots, \alpha_{MS}) \quad \alpha_{ms} \geq 1\ \forall\ m, s \tag{6}$$

and for the analyses presented here we use a uniform over the simplex, i.e. $\alpha_{ms} \equiv 1$.

Here we derive an EM algorithm to maximize the log posterior for $(\boldsymbol{\Sigma}_{1:S}, \boldsymbol{\pi})$ under the model specified at (1)-(6), i.e. to find

$$\hat{\boldsymbol{\Sigma}}_{1:S}, \hat{\boldsymbol{\pi}} := \underset{\boldsymbol{\Sigma}_{1:S}, \boldsymbol{\pi}}{\operatorname{argmax}}\ \log p(\boldsymbol{\Sigma}_{1:S}, \boldsymbol{\pi} \mid \hat{\boldsymbol{\Theta}}^{\mathrm{UV}}, \hat{\boldsymbol{R}})$$

or rewritten as the sum of log likelihood and log prior:

$$\hat{\boldsymbol{\Sigma}}_{1:S}, \hat{\boldsymbol{\pi}} = \underset{\boldsymbol{\Sigma}_{1:S}, \boldsymbol{\pi}}{\operatorname{argmax}}\ \log p(\hat{\boldsymbol{\Theta}}^{\mathrm{UV}} \mid \boldsymbol{\Sigma}_{1:S}, \boldsymbol{\pi}, \hat{\boldsymbol{R}}) + \log[p(\boldsymbol{\Sigma}_{1:S}) p(\boldsymbol{\pi})]\ . \tag{7}$$

In the context of the EM algorithm,

$\mathcal{D} := \hat{\theta}^{\mathrm{UV}}_{1:P,1:G}$ are observed data,
$\mathcal{M} := \{\theta_{1:P,1:G}, m_{1:G}, s_{1:G}\}$ are latent quantities, and
$\mathcal{P} := \{\boldsymbol{\Sigma}_{1:S}, \pi_{1:M,1:S}\}$ are model parameters.

Note that in $\mathcal{M}$ we have introduced $m_{1:G}$ and $s_{1:G}$ as the latent variables defining membership of mixture components, i.e.

$$\boldsymbol{\theta}_{\cdot g} \mid \boldsymbol{\Sigma}_s, m_g = m, s_g = s \ \sim\ \mathrm{N}(\boldsymbol{0},\ \omega_m \boldsymbol{\Sigma}_s)$$

and that *a priori*:

$$\mathbb{P}(m_g = m, s_g = s \mid \pi_{ms}) = \pi_{ms}\ .$$

Note also that $\hat{\boldsymbol{R}}$ is estimated in advance from samples distributed according to the experimental noise process without biological signal; in the case of the International Mouse Phenotyping Consortium (IMPC) data, we randomly sub-sample wild-type animals to create synthetic null data, as described in Methods–*Control of error rates.*

The optimization at (7), rewritten in the $\mathcal{D}, \mathcal{M}, \mathcal{P}$ notation above is

$$\hat{\mathcal{P}} = \underset{\mathcal{P}}{\operatorname{argmax}}\ \log p(\mathcal{D} \mid \mathcal{P}) + \log p(\mathcal{P}) \tag{8}$$

and the E- and M-steps of the EM algorithm applied iteratively for optimizing (8) are, respectively,

$$\begin{aligned} Q(\mathcal{P};\mathcal{P}^{(t)}) &:= \mathbb{E}_{\mathcal{M}|\mathcal{D},\mathcal{P}^{(t)}}[\log p(\mathcal{M},\mathcal{D} \mid \mathcal{P})] && (9)\\ \mathcal{P}^{(t+1)} &= \underset{\mathcal{P}}{\operatorname{argmax}} \ Q(\mathcal{P};\mathcal{P}^{(t)}) + \log p(\mathcal{P}) \ . && (10) \end{aligned}$$

0.1 E-step

In this stage we evaluate

$$Q(\mathcal{P};\mathcal{P}^{(t)}) \quad := \quad \mathrm{E}_{\mathcal{M}|\mathcal{D},\mathcal{P}^{(t)}}[\log p(\mathcal{M},\mathcal{D} \mid \mathcal{P})]$$

proceeding as follows:

$$\begin{aligned} &Q(\mathcal{P};\mathcal{P}^{(t)})\\ &= \mathbb{E}_{\mathcal{M}|\mathcal{D},\mathcal{P}^{(t)}} \log p(\mathcal{M},\mathcal{D} \mid \mathcal{P})\\ &= \sum_{g=1}^{G} \mathbb{E}_{\boldsymbol{\theta}_{\cdot g},m_g,s_g|\mathcal{D}_g,\mathcal{P}^{(t)}} \log p(\boldsymbol{\theta}_{\cdot g}, m_g, s_g, \mathcal{D}_g \mid \mathcal{P})\\ &= \sum_{g} \mathbb{E}_{\boldsymbol{\theta}_{\cdot g},m_g,s_g|\mathcal{D}_g,\mathcal{P}^{(t)}} \log \prod_{m,s} [\mathbb{P}(m_g=m, s_g=s \mid \boldsymbol{\pi}) p(\boldsymbol{\theta}_{\cdot g}, \mathcal{D}_g \mid \mathcal{P}, m_g=m, s_g=s)]^{\mathbb{I}(m_g=m,s_g=s)}\\ &= \sum_{g,m,s} \mathbb{E}_{\boldsymbol{\theta}_{\cdot g},m_g,s_g|\mathcal{D}_g,\mathcal{P}^{(t)}} [\mathbb{I}(m_g=m,s_g=s)\log(\pi_{ms} p(\boldsymbol{\theta}_{\cdot g}, \mathcal{D}_g \mid \mathcal{P}, m_g=m, s_g=s))]\\ &= \sum_{g,m,s} \mathbb{E}_{m_g,s_g|\mathcal{D}_g,\mathcal{P}^{(t)}} \mathbb{E}_{\boldsymbol{\theta}_{\cdot g}|m_g,s_g\mathcal{D}_g,\mathcal{P}^{(t)}} [\mathbb{I}(m_g=m,s_g=s)\log(\pi_{ms} p(\boldsymbol{\theta}_{\cdot g}, \mathcal{D}_g \mid \mathcal{P}, m_g=m, s_g=s))]\\ &= \sum_{g,m,s} \mathbb{E}_{m_g,s_g|\mathcal{D}_g,\mathcal{P}^{(t)}} \Big[\mathbb{I}(m_g=m,s_g=s) \mathbb{E}_{\boldsymbol{\theta}_{\cdot g}|m_g,s_g\mathcal{D}_g,\mathcal{P}^{(t)}} \log(\pi_{ms} p(\boldsymbol{\theta}_{\cdot g}, \mathcal{D}_g \mid \mathcal{P}, m_g=m, s_g=s))\Big]\\ &= \sum_{g,m,s} \mathbb{P}(m_g=m,s_g=s \mid \mathcal{D}_g, \mathcal{P}^{(t)}) \mathbb{E}_{\boldsymbol{\theta}_{\cdot g}|\mathcal{D}_g,\mathcal{P}^{(t)},m_g=m,s_g=s} \log(\pi_{ms} p(\boldsymbol{\theta}_{\cdot g}, \mathcal{D}_g \mid \mathcal{P}, m_g=m, s_g=s))\\ &= \sum_{g,m,s} r_{gms}^{(t)} \Big(\log \pi_{ms} + \mathbb{E}_{\boldsymbol{\theta}_{\cdot g}|\mathcal{D}_g,\mathcal{P}^{(t)},m_g=m,s_g=s} \log p(\boldsymbol{\theta}_{\cdot g}, \mathcal{D}_g \mid \mathcal{P}, m_g=m, s_g=s)\Big)\\ &= \sum_{g,m,s} r_{gms}^{(t)} \Big(\log \pi_{ms} + \xi_{gms}^{(t)}\Big) \qquad (11) \end{aligned}$$

where

$$\begin{aligned} r_{gms}^{(t)} &:= \mathbb{P}(m_g=m, s_g=s \mid \hat{\boldsymbol{\theta}}_{*g}^{\mathrm{UV}}, \mathcal{P}^{(t)})\\ &= \frac{\mathbb{P}(\hat{\boldsymbol{\theta}}_{*g}^{\mathrm{UV}} \mid m_g=m, s_g=s, \mathcal{P}^{(t)})\pi_{ms}^{(t)}}{\sum_{m,s} \mathbb{P}(\hat{\boldsymbol{\theta}}_{*g}^{\mathrm{UV}} \mid m_g=m, s_g=s, \mathcal{P}^{(t)})\pi_{ms}^{(t)}} \end{aligned}$$

and

$$\xi_{gms}^{(t)} \quad := \quad \mathbb{E}_{\boldsymbol{\theta}_{\cdot g}|\hat{\boldsymbol{\theta}}_{*g}^{\mathrm{UV}},\mathcal{P}^{(t)},m_g=m,s_g=s} \log p(\boldsymbol{\theta}_{\cdot g}, \hat{\boldsymbol{\theta}}_{*g}^{\mathrm{UV}} \mid \mathcal{P}, m_g=m, s_g=s) \ . \qquad (12)$$

To derive $\xi_{gms}^{(t)}$ we first note that

$$\begin{aligned} &\log p(\boldsymbol{\theta}_{\cdot g}, \hat{\boldsymbol{\theta}}_{*g}^{\mathrm{UV}} \mid \mathcal{P}, m_g=m, s_g=s)\\ &= \log p(\boldsymbol{\theta}_{\cdot g} \mid \mathcal{P}, m_g=m, s_g=s) + \log p(\hat{\boldsymbol{\theta}}_{*g}^{\mathrm{UV}} \mid \boldsymbol{\theta}_{\cdot g}, \mathcal{P}, m_g=m, s_g=s)\\ &= \log \mathrm{N}(\boldsymbol{\theta}_{\cdot g} \mid \mathbf{0}, \omega_m \boldsymbol{\Sigma}_s) + \log \mathrm{N}(\hat{\boldsymbol{\theta}}_{*g}^{\mathrm{UV}} \mid \boldsymbol{\theta}_{*g}, \hat{\boldsymbol{S}}_{**g}^{\mathrm{UV}} \hat{\boldsymbol{R}}_{**} \hat{\boldsymbol{S}}_{**g}^{\mathrm{UV}})\\ &\overset{\boldsymbol{\Sigma}_s}{=} -\Big(\log|\boldsymbol{\Sigma}_s| + \boldsymbol{\theta}_{\cdot g}^T (\omega_m \boldsymbol{\Sigma}_s)^{-1} \boldsymbol{\theta}_{\cdot g}\Big)/2 \ . \qquad (13) \end{aligned}$$

We then target the distribution for the expectation operator $\mathbb{E}_{\boldsymbol{\theta}_{\cdot g}|\hat{\boldsymbol{\theta}}_{*g}^{\mathrm{UV}},\mathcal{P}^{(t)},m_g=m,s_g=s}$ in $\xi_{gms}^{(t)}$, noting the following joint conditional distribution for $\boldsymbol{\theta}_{\cdot g}$ and $\hat{\boldsymbol{\theta}}_{*g}^{\mathrm{UV}}$:

$$\begin{bmatrix} \boldsymbol{\theta}_{\cdot g} \\ \hat{\boldsymbol{\theta}}_{*g}^{\mathrm{UV}} \end{bmatrix} \mid \mathcal{P}^{(t)}, m_g=m, s_g=s \quad \sim \quad \mathrm{N}\left(\mathbf{0}, \begin{bmatrix} \omega_m \boldsymbol{\Sigma}_s^{(t)} & \omega_m \boldsymbol{\Sigma}_{\cdot * s}^{(t)} \\ \omega_m \boldsymbol{\Sigma}_{* \cdot s}^{(t)} & \omega_m \boldsymbol{\Sigma}_{**s}^{(t)} + \hat{\boldsymbol{S}}_{g,**}^{\mathrm{UV}} \hat{\boldsymbol{R}}_{**} \hat{\boldsymbol{S}}_{g,**}^{\mathrm{UV}} \end{bmatrix}\right) \qquad (14)$$

so that the conditional distribution $\boldsymbol{\theta}_{\cdot g} \mid \hat{\boldsymbol{\theta}}^{\text{UV}}_{*g}$ follows from the conditional multivariate Gaussian identity:

$$
\begin{aligned}
\boldsymbol{\theta}_{\cdot g} \mid \hat{\boldsymbol{\theta}}^{\text{UV}}_{*g}, \mathcal{P}^{(t)}, m_g = m, s_g = s \quad &\sim \quad \mathrm{N}(\boldsymbol{\mu}^{(t)}_{gms}, \boldsymbol{V}^{(t)}_{gms}) \\
\boldsymbol{\mu}^{(t)}_{gms} \quad &:= \quad \omega_m \boldsymbol{\Sigma}^{(t)}_{s,\cdot *} \left(\omega_m \boldsymbol{\Sigma}^{(t)}_{**s} + \hat{\boldsymbol{S}}^{\text{UV}}_{g,**} \hat{\boldsymbol{R}}_{**} \hat{\boldsymbol{S}}^{\text{UV}}_{g,**} \right)^{-1} \hat{\boldsymbol{\theta}}^{\text{UV}}_{*g} \\
\boldsymbol{V}^{(t)}_{gms} \quad &:= \quad \omega_m \boldsymbol{\Sigma}^{(t)}_{s,\cdot\cdot} - \omega_m \boldsymbol{\Sigma}^{(t)}_{s,\cdot *} \left(\omega_m \boldsymbol{\Sigma}^{(t)}_{**s} + \hat{\boldsymbol{S}}^{\text{UV}}_{g,**} \hat{\boldsymbol{R}}_{**} \hat{\boldsymbol{S}}^{\text{UV}}_{g,**} \right)^{-1} \omega_m \boldsymbol{\Sigma}^{(t)}_{s,*\cdot}
\end{aligned}
\tag{15}
$$

Combining (13) and (15) in (12) gives

$$
\begin{aligned}
\xi^{(t)}_{gms} \quad &\overset{\boldsymbol{\Sigma}_s}{=} \quad -\frac{1}{2} \mathbb{E}_{\boldsymbol{\theta}_{\cdot g} \mid \hat{\boldsymbol{\theta}}^{\text{UV}}_{*g}, \mathcal{P}^{(t)}, m_g = m, s_g = s} \left(\log |\boldsymbol{\Sigma}_s| + \boldsymbol{\theta}^T_{\cdot g} (\omega_m \boldsymbol{\Sigma}_s)^{-1} \boldsymbol{\theta}_{\cdot g} \right) \\
&= \quad -\frac{1}{2} \log |\boldsymbol{\Sigma}_s| - \frac{1}{2} \mathrm{tr} \left[(\omega_m \boldsymbol{\Sigma}_s)^{-1} (\boldsymbol{V}^{(t)}_{gms} + \boldsymbol{\mu}^{(t)}_{gms} \boldsymbol{\mu}^{(t)T}_{gms}) \right] .
\end{aligned}
\tag{16}
$$

Substituting (16) into (11) gives

$$
Q(\mathcal{P}; \mathcal{P}^{(t)}) \quad \overset{\boldsymbol{\Sigma}, \boldsymbol{\pi}}{=} \quad \sum_{g,m,s} r^{(t)}_{gms} \left(\log(\pi_{ms}) - \frac{1}{2} \log |\boldsymbol{\Sigma}_s| - \frac{1}{2} \mathrm{tr} \left[(\omega_m \boldsymbol{\Sigma}_s)^{-1} (\boldsymbol{V}^{(t)}_{gms} + \boldsymbol{\mu}^{(t)}_{gms} \boldsymbol{\mu}^{(t)^T}_{gms}) \right] \right)
\tag{17}
$$

0.2 M-step

In this stage we compute $\boldsymbol{\Sigma}^{(t+1)}_s, \pi^{(t+1)}$, as per the M-step:

$$
\mathcal{P}^{(t+1)} \quad = \quad \underset{\mathcal{P}}{\operatorname{argmax}} \;\; Q(\mathcal{P}; \mathcal{P}^{(t)}) + \log p(\mathcal{P}) \; .
$$

The optimisation can performed on $\boldsymbol{\Sigma}^{(t+1)}_s$ separately for each s; likewise, $\pi^{(t+1)}$ can be optimised separately.

Addressing first the optimisation with respect to $\boldsymbol{\Sigma}^{(t+1)}_s$ for one particular s, collecting relevant terms from (17) and from the priors at (4) and (6) gives:

$$
\boldsymbol{\Sigma}^{(t+1)}_s \quad = \quad \underset{\boldsymbol{\Sigma}}{\operatorname{argmax}} \left\{ \log |\boldsymbol{\Sigma}| + \mathrm{tr}(\boldsymbol{\Sigma}^{-1} \boldsymbol{C}^{(t)}_s) : \boldsymbol{\Sigma} \in \mathcal{W}_K \right\}
\tag{18}
$$

$$
\mathcal{W}_K \quad := \quad \{ \boldsymbol{W} \boldsymbol{W}^T + \boldsymbol{\Psi}_s : \boldsymbol{W} \in \mathbb{R}^{P \times K}, \;\; \boldsymbol{\Psi}_s \text{ diagonal with } [\boldsymbol{\Psi}_s]_{jj} \geq 0 \; \forall j \}
\tag{19}
$$

where

$$
\boldsymbol{C}^{(t)}_s := \frac{\sum_{g,m} r^{(t)}_{gms} (\boldsymbol{V}^{(t)}_{gms} + \boldsymbol{\mu}^{(t)}_{gms} \boldsymbol{\mu}^{(t)^T}_{gms}) / \omega_m}{\sum_{g,m} r^{(t)}_{gms}} \; .
\tag{20}
$$

The optimisation at (18)-(19) can be performed using standard factor analysis software (we use `stats::factanal()` in R).

Finally, to maximize $Q(\mathcal{P}; \mathcal{P}^{(t)})$ with respect to $\boldsymbol{\pi}$ we find

$$
\boldsymbol{\pi}^{(t+1)} \quad = \quad \underset{\boldsymbol{\pi} \in \mathcal{S}}{\operatorname{argmax}} \sum_{m,s} (\tilde{\alpha}_{ms} - 1) \log \pi_{ms}
\tag{21}
$$

$$
\tilde{\alpha}_{ms} \quad := \quad \alpha_{ms} + \sum_g r^{(t)}_{gms}
\tag{22}
$$

$$
\mathcal{S} \quad := \quad \left\{ \pi_{1:M,1:S} : \sum_{m,s} \pi_{ms} = 1, \;\; \pi_{ms} \geq 0 \right\}
$$

and the objective in (21) has the form of a Dirichlet$(\tilde{\alpha}_{11}, \ldots, \tilde{\alpha}_{MS})$ on vec$(\boldsymbol{\pi})$, having the unique mode

$$
\pi^{(t+1)}_{ms} \quad = \quad \frac{\tilde{\alpha}_{ms} - 1}{\sum_{m,s} (\tilde{\alpha}_{ms} - 1)}
\tag{23}
$$

provided that $\tilde{\alpha}_{ms} \geq 1 \, \forall \, m, s$ (and this is implied by the prior on $\boldsymbol{\pi}$ at (6) satisfying $\alpha_{ms} \geq 1 \, \forall \, m, s$). In the case of a uniform prior having $\alpha_{ms} \equiv 1$, the update is (with reference to (22) and (23))

$$\pi_{ms}^{(t+1)} = \frac{\sum_g r_{gms}^{(t)}}{\sum_{g,m,s} r_{gms}^{(t)}} . \tag{24}$$

References

[1] Bovy, J., Hogg, D. W. & Roweis, S. T. Extreme deconvolution: Inferring complete distribution functions from noisy, heterogeneous and incomplete observations. *Annals of Applied Statistics* **5**, 1657–1677 (2011).

[2] Urbut, S. M., Wang, G., Carbonetto, P. & Stephens, M. Flexible statistical methods for estimating and testing effects in genomic studies with multiple conditions. *Nature Genetics* **51**, 187–195 (2019). URL `http://www.nature.com/articles/s41588-018-0268-8`.

[3] Bishop, C. *Pattern Recognition and Machine Learning | Christopher Bishop | Springer* (Springer-Verlag New York, 2006), 1 edn. URL `https://www.springer.com/gp/book/9780387310732`.
