# Supplementary figures and images for: Multivariate phenotype analysis enables genome-wide inference of mammalian gene function
Source: PLoS Biol. 2022 Aug 9;20(8):e3001723. doi: 10.1371/journal.pbio.3001723 (PMC9391051; doi:10.1371/journal.pbio.3001723)

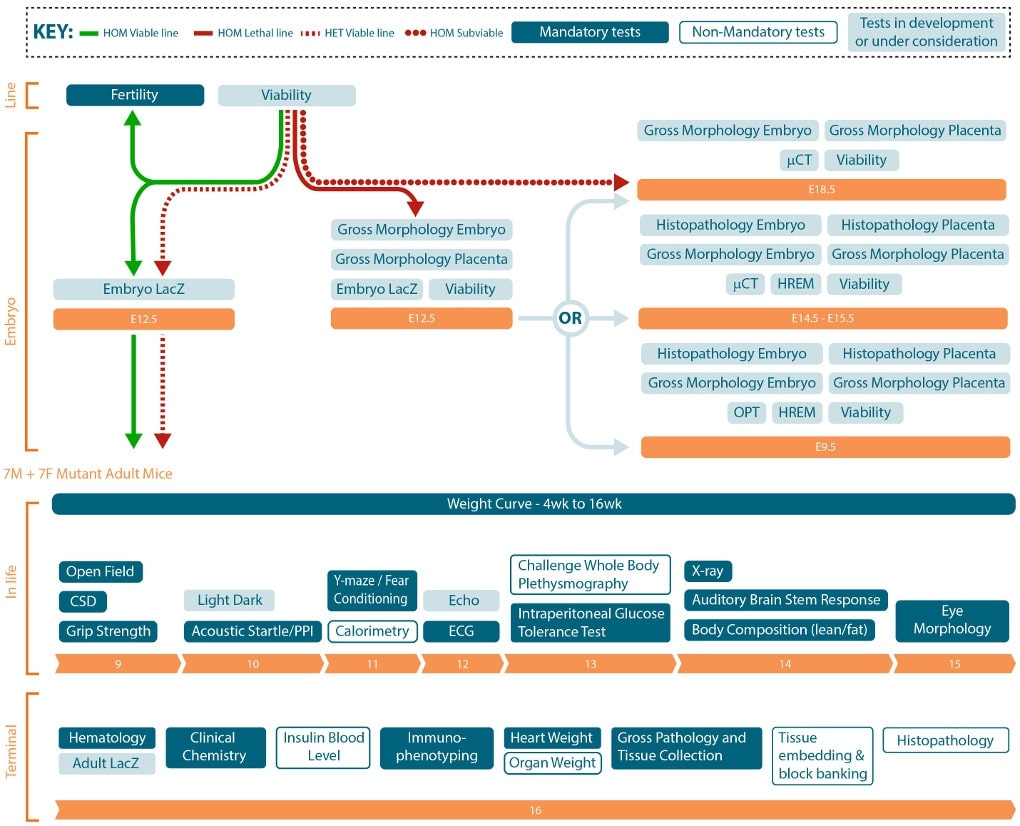

Supplement: S1 Fig — Scientific purpose, experimental design, and detailed description for each procedure are available at www.mousephenotype.org/impress/pipelines. Each phenotype within each procedure is also described in detail. Note that the terminology parameters is used there to refer to what we call phenotypes in this paper. We prefer to use phenotypes to avoid any terminological ambiguity with the use of parameters in statistical inference. (TIF) [file pbio.3001723.s003.tif]

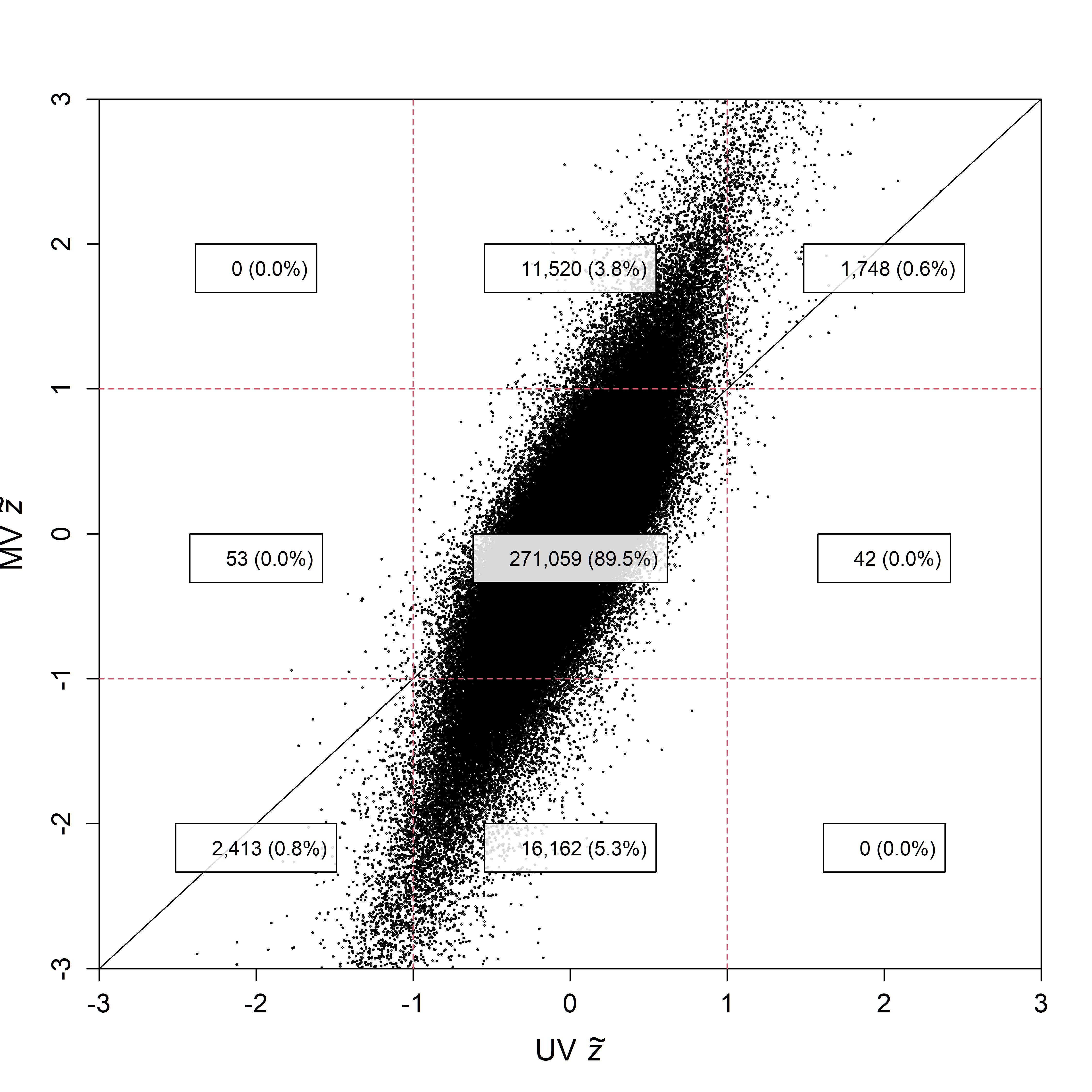

Supplement: S3 Fig — The axes extend to [−3, 3] while the counts apply to all data, including those beyond the scale of the plot. The data and code used to generate this figure are available at [13,14]. MV, multivariate; UV, univariate. (TIF) [file pbio.3001723.s005.tif]

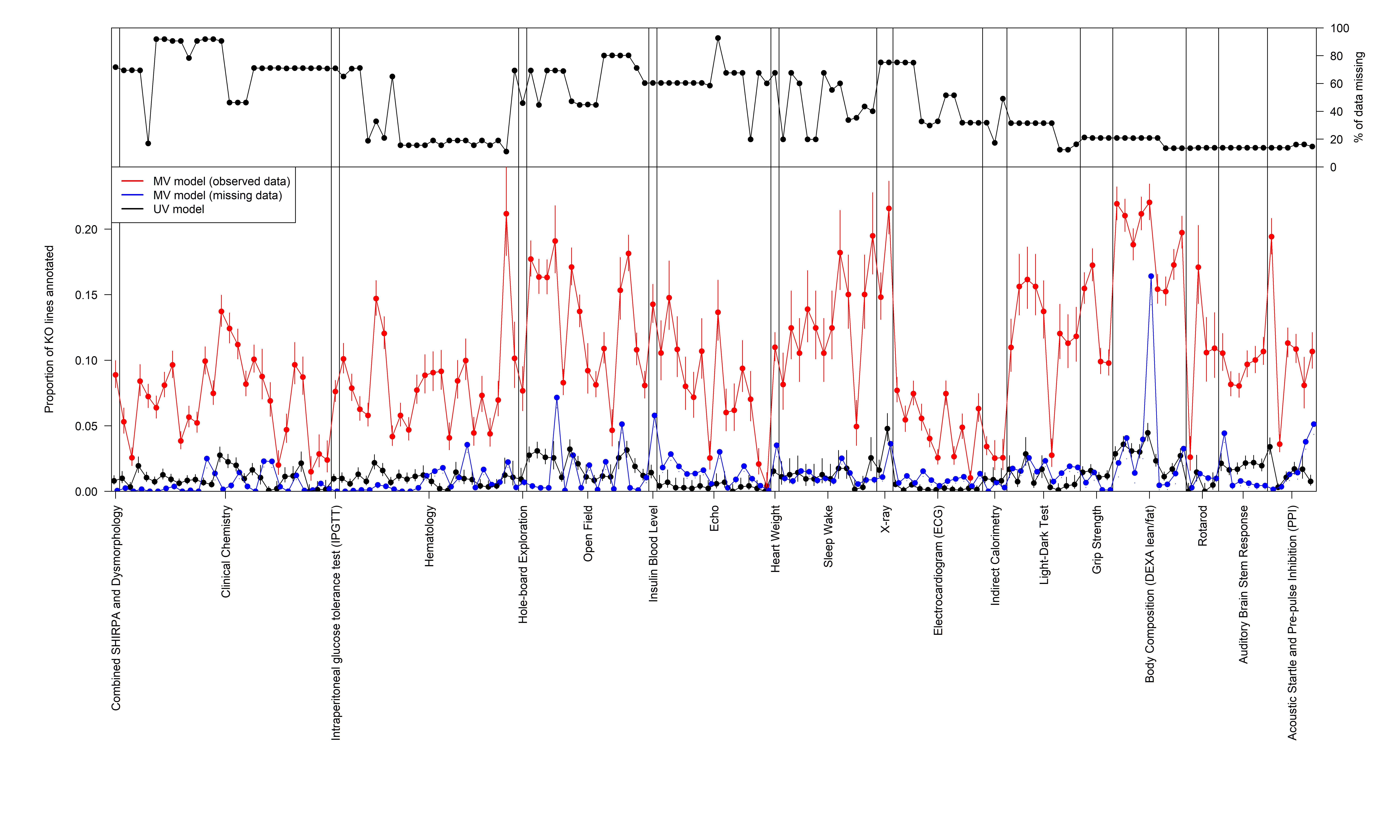

Supplement: S4 Fig — The top panel shows the % missing data for each phenotype. The lower panel displays the phenotype-specific hit rate (i.e., proportion of lines that are significantly perturbed), for the UV method, and for the MV method stratified according to whether data are missing or observed. The data and code used to generate this figure are available at [13,14]. MV, multivariate; UV, univariate. (TIF) [file pbio.3001723.s006.tif]

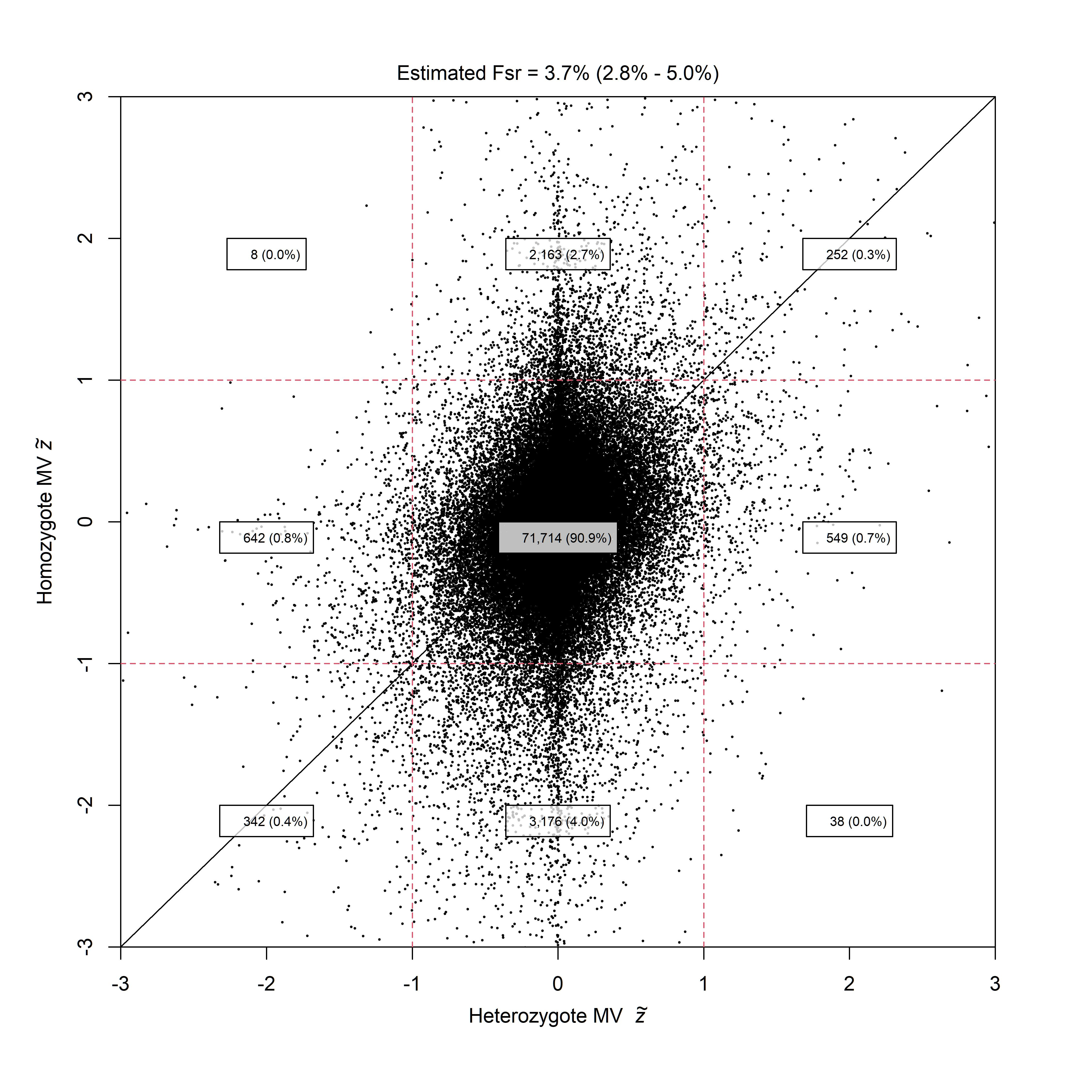

Supplement: S6 Fig — Each point corresponds to the z˜pg of the heterozygote and homozygote KO lines of a particular gene. Counts (%) for each significance combination are superimposed; while the axes extend to [−3, 3], the counts apply to all data, including those beyond the plot’s scale. An Fsr estimate Fsr^replicate (95% CI) based on the level of discordance is shown at the top of the panel. The data and code used to generate this figure are available at [13,14]. Fsr, false sign rate; KO, knockout. (TIF) [file pbio.3001723.s008.tif]

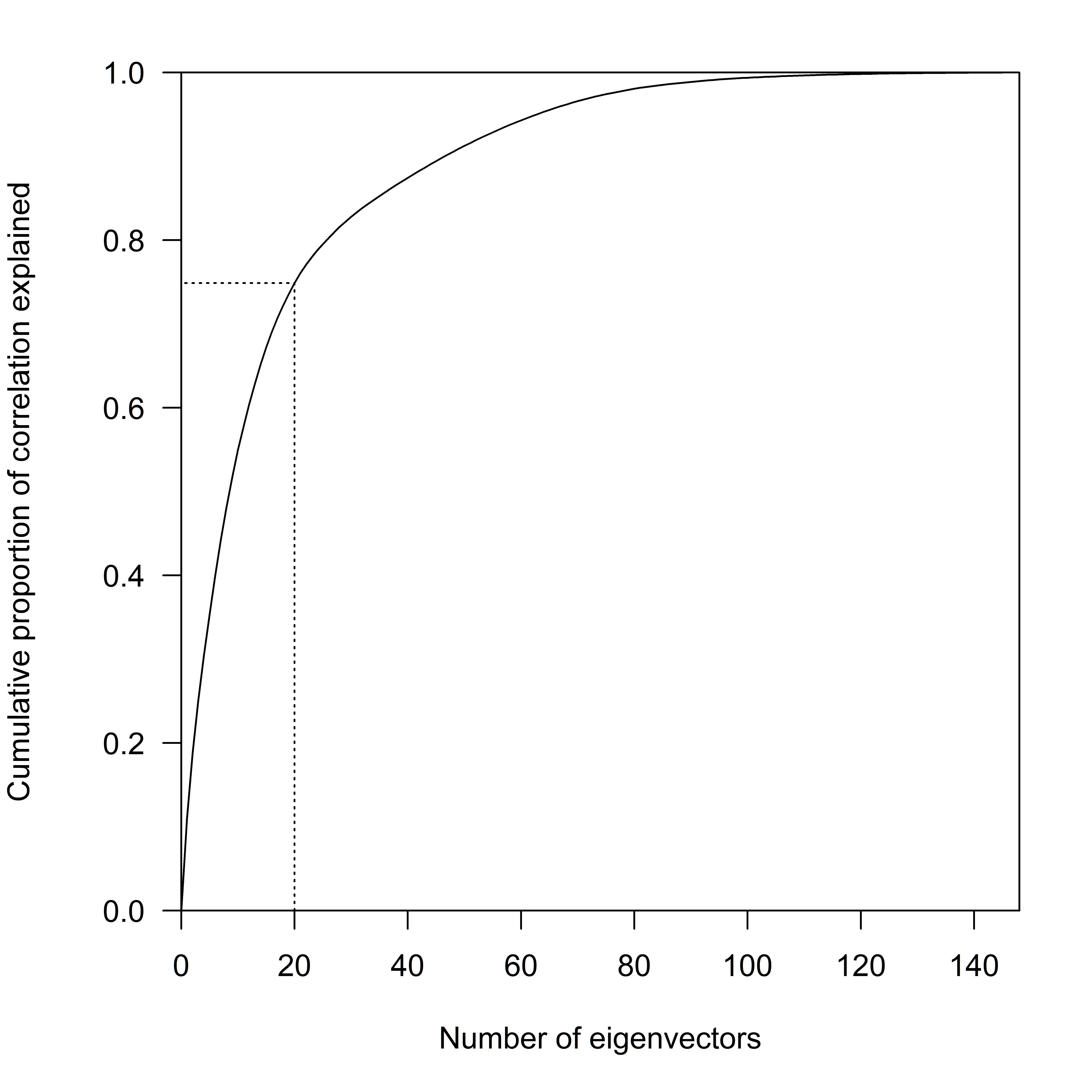

Supplement: S8 Fig — The dotted line indicates that over 75% of the correlation is explained by 20 eigenvectors. The data and code used to generate this figure are available at [13,14]. (TIF) [file pbio.3001723.s010.tif]

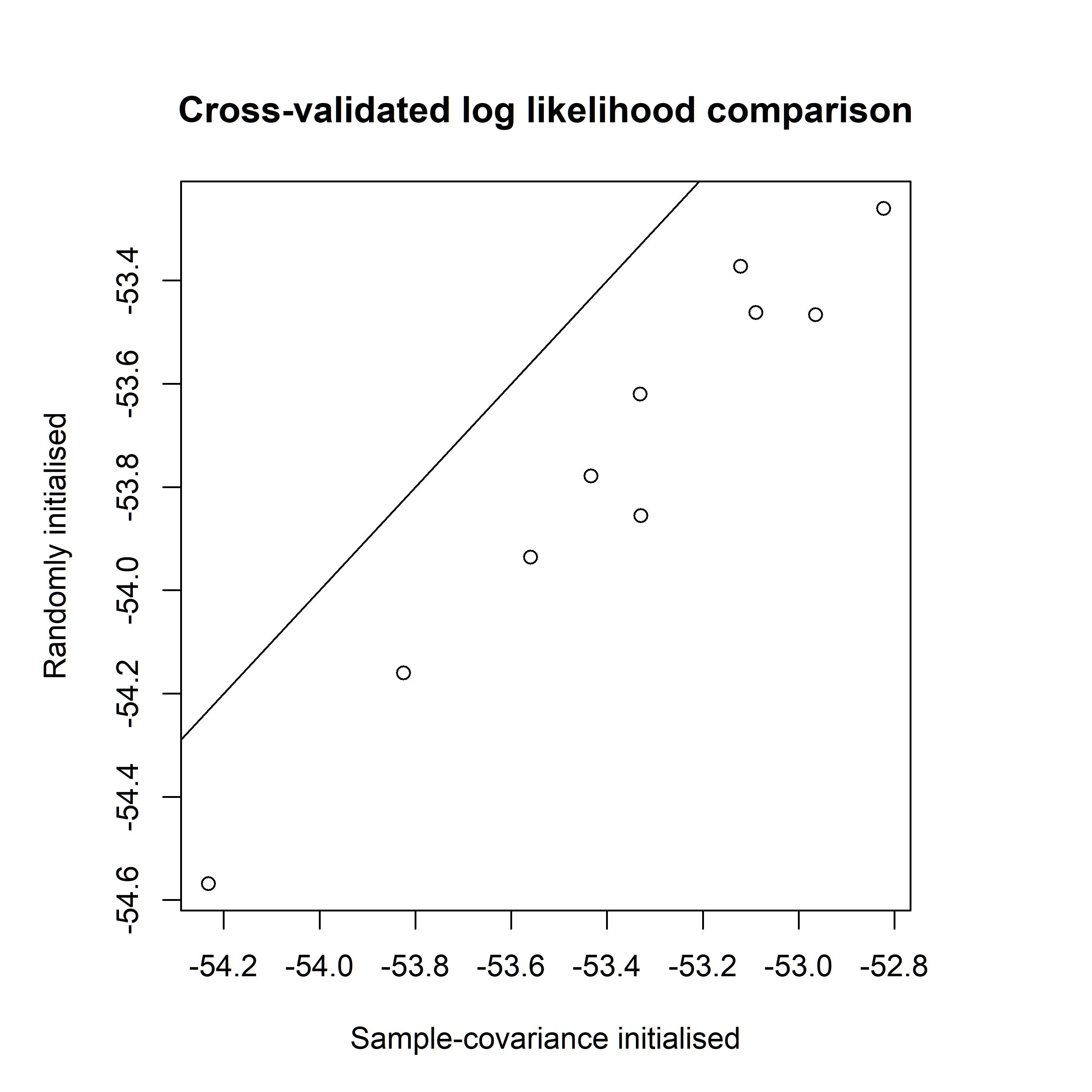

Supplement: S9 Fig — We plot LOO-MV results (inferring perturbations on masked data) against results for the UV model applied to the unmasked data; see Methods–Predicting masked data. An Fsr estimate Fsr^replicate (95% CI) based on the level of discordance is shown at the top of the panel. The data and code used to generate this figure are available at [13,14]. Fsr, false sign rate; LOO-MV, leave-one-procedure-out MV; MV, multivariate; UV, univariate. (TIF) [file pbio.3001723.s011.tif]

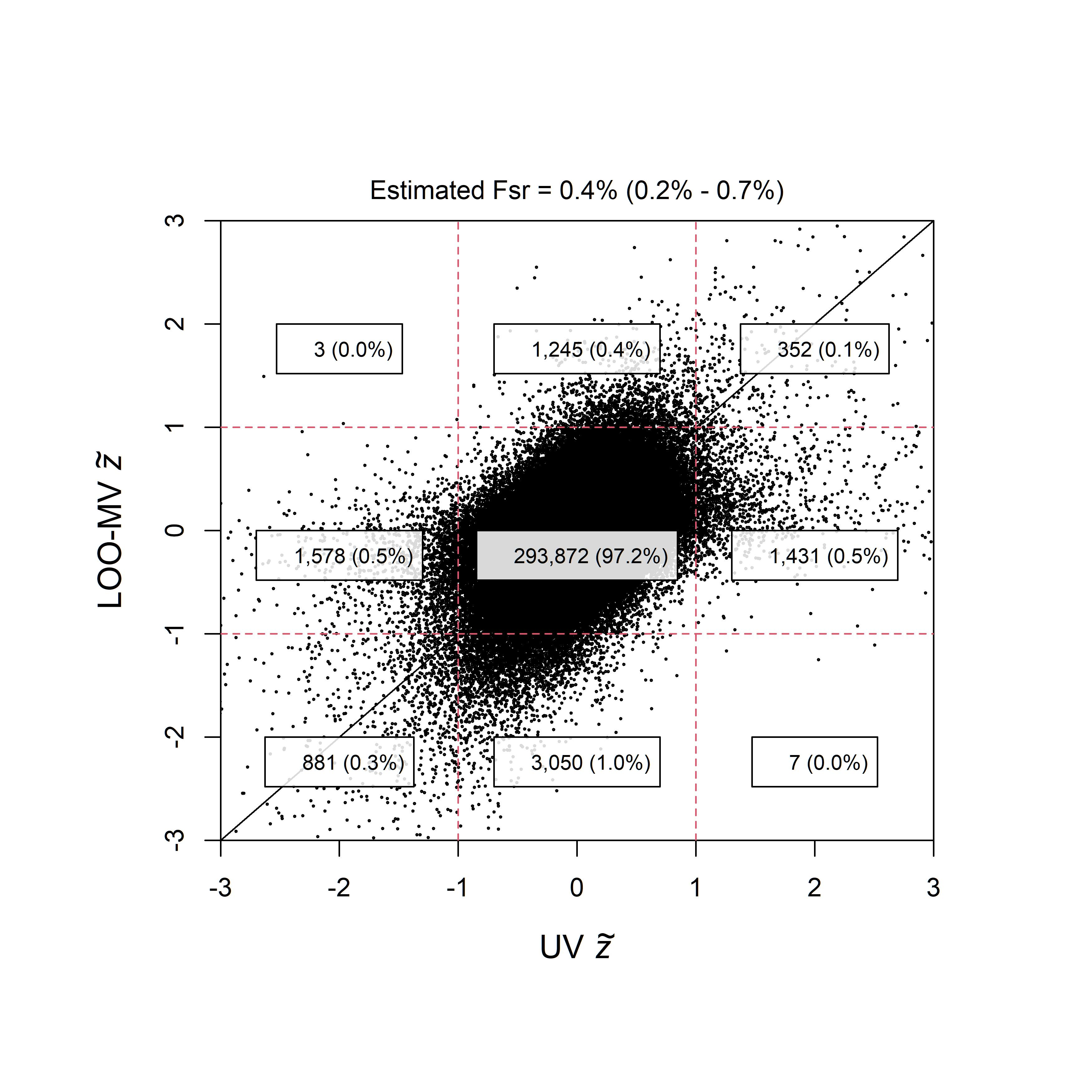

Supplement: S11 Fig — (a) The varimax-rotated loadings for Σ^pooled, the Bayesian model averaged covariance matrix across all cross-validation folds. (b) The varimax-rotated loadings for the fold c′ covariance matrix Σ^(c′), which is chosen to maximise the symmetrized KL divergence between N(0,Σ^pooled) and N(0,Σ^(c)) across folds c. The 2 loadings plots are qualitatively similar, though there are some small discrepancies. The data and code used to generate this figure are available at [13,14]. (TIF) [file pbio.3001723.s013.tif]
