# Supplementary material for: Multivariate phenotype analysis enables genome-wide inference of mammalian gene function
Source: PLoS Biol. 2022 Aug 9;20(8):e3001723. doi: 10.1371/journal.pbio.3001723 (PMC9391051; doi:10.1371/journal.pbio.3001723)
Supplement: Attachment — Submitted filename: response_to_reviewers.pdf [file pbio.3001723.s014.pdf]

Oxford, 18th March 2022

Dear Reviewers,

Thank you so much for your kind generosity in dedicating your valuable time and thoughtful energy to peer reviewing our work.

Two years have now elapsed since you reviewed our paper, and we would like to say sorry for our delay in replying. Please forgive us—many of our co-authors on this paper have been extremely focused on the COVID-19 pandemic over the past year and a half.[3]

Your excellent comments have motivated us greatly to improve the paper. A few of the major changes are:

1. We introduce an enhanced multivariate modelling framework that builds on existing work in this area, incorporates many of the improvements that you suggested—including a factor representation, and a mixture model allowing covariance to vary across KO lines—and introduces a cross-validation step to combat any over-fitting.
2. We benchmark our method against mash (Urbut et al. [20]) as you suggested and also against other related work known as extreme deconvolution (Bovy et al. [21]); this demonstrates that our method performs favourably compared to existing work, while explicitly building upon it.
3. We have written a new section examining co-enrichment between IMPC phenotypes and gene ontology terms. This adds an interesting new biological facet to the paper, demonstrates the benefits of the increased power of the MV approach relative to its UV counterpart, and acts as an extra form of biological validation, given that we see scientifically sensible results emerging.

These are just the major changes, and we have spent a lot of time on several other aspects as well, trying to give every point that you have made the attention it deserves.

We hope you like the improved version of our paper!

Yours faithfully,

George Nicholson (on behalf of all co-authors)

[3] Here is a flavour of what we have been working on:

- `https://www.nature.com/articles/s41564-021-01029-0`
- `https://arxiv.org/abs/2109.13730`
- `https://arxiv.org/abs/2103.12661`
- `https://www.thelancet.com/journals/lanepe/article/PIIS2666-7762(22)00015-1/fulltext`

While this work is not relevant to the current paper, we hope this mitigates our slow response to your comments!

Reviewer 1

Introduction

This MS describes a sophisticated multivariate analysis of over 6,000 IMPC gene knockouts measured on 148 phenotypes. The analysis aims to call correctly the phenotypic effect for each gene on each phenotype at a controlled false discovery rate, and to impute missing results where possible. This dataset is difficult to work with because of its internal structure with multiple levels of correlation.

The analysis methodology is a hybrid of Bayesian and Frequentist steps, which makes it rather hard to follow in places. While I don't have any substantive concerns (other than those noted below) one is left with the feeling that the pipeline is over-complicated. I think this is mainly because the MV analysis has been bolted onto the outputs of the UV analysis (author reply: see P 1.1 below). Nonetheless, the results are encouraging, in that a larger fraction of positive phenotype calls are made using multivariate analysis compared to univariate analysis, whilst controlling the Fdr. As far as I can tell, without redoing the analysis, the results are correct, and the models fitted are sensible. This alone is a very significant achievement.

Major comments

Reviewer Point P 1.1 — While I don't have any substantive concerns (other than those noted below) one is left with the feeling that the pipeline is over-complicated. I think this is mainly because the MV analysis has been bolted onto the outputs of the UV analysis.

Reply: Thanks for this helpful observation. We have now placed our current work into better context against the background of established research, building on to existing methods, extreme deconvolution and mash [21, 20], which introduced the idea of two-stage inference performing MV analysis on outputs from UV analysis. We have also now done some benchmarking and qualitative methods comparison between the current MV approach and existing methods (Section 5.6). We agree that the pipeline is a little complex – we do feel that each stage is vital to the high power under careful error rate control that the method attains. We have tried to lay out the rationale for and clarify the method in a number of places, particularly in 5.3, and we have included extra model validation in Section 5.4.

Reviewer Point P 1.2 — There are many important methodological details and caveats which are buried in the methods, and which makes the MS hard to follow.

Reply: Great point, we have moved the details of the UV and MV models to the start of Results (Sections 3.1 and 3.2) and we hope that this makes the manuscript easier to follow.

Reviewer Point P 1.3 — The MS has a large number of figures (particularly heatmaps) which are not always very informative. The authors should review carefully whether all these figures are necessary or could be moved to the supplement.

Reply: Yes, we have done this and have moved the reference lines replicability heatmap to the SI, as Supplementary Figure 5.

Reviewer Point P 1.4 — Some simple pie charts, or similar, giving the fractions of positive phenotype calls, sex effects, how much the imputation increased the fraction of positive calls etc, would be helpful additions.

Reply: Thanks, nice idea, we have now added Venn diagrams in Figure 4 that we hope encapsulate the increased hit rate on missing and observed data, as well as the overlap between different methods.

Reviewer Point P 1.5 — This is not meant to detract from the considerable achievement of a generally excellent statistical analysis, but I wonder what a biologist will make of the findings. For instance, there is no attempt to use the increased phenotype call rate to make inferences about

which gene sets are involved in phenotypes, and if these sets are related to known gene networks, or possibly physically clustered on the genome.

Reply: Thanks for the kind words and for the great suggestion. We have now added a section investigating coenrichment between GO-term gene sets and the gene sets perturbed by each phenotype (Section 3.8). This section gives us a glimpse of the tremendous biological insights that will hopefully be available once the IMPC database is complete. Also, the reasonable nature of the (GO-term, IMPC phenotype) co-enrichment is an extra form of validation that the MV method is performing well.

Reviewer Point P 1.6 — Finally, what is the status of these new phenotype calls - will they replace the existing IMPC annotations?

Reply: Good question. There is definitely a willingness among the senior leadership team of the IMPC to introduce the MV approach (including inference on missing data) into a parallel analysis made available for the moment alongside the current analytical tools and output.

Reviewer Point P 1.7 — How do the positive gene annotations generated in the current MS differ from those reported elsewhere?

Reply: We compared the gene annotations from our UV and MV models to the existing IMPC annotations and observe a very low degree of disagreement, and with the MV model having higher power as is common with multivariate vs. univariate methods. We have included the comparison in a new section, 3.6.

Reviewer Point P 1.8 — What is the desired end point: is this a methods paper, or the definitive IMPC analysis?

Reply: Good question. The answer has actually become much clearer over the course of revising the paper, and we believe we can safely say that this paper has contributions to make in both areas.

In terms of methods development, the methodology in the revised version is very much oriented as building on existing methods [21, 20]. We include a performance comparison that shows our methodological extensions yield higher performance according to metrics such as better hit rates, replicability and cross-validated likelihoods (please see Methods–*Comparison with existing methods*).

In terms of how this new work can be used to augment the existing IMPC pipeline, it can contribute in terms of identifying phenotypic perturbations with higher power, and can identify effects the reasonable power even when the phenotype data are missing, both of which have the potential to strengthen the eventual impact of the IMPC.

Technical points requiring clarification

Reviewer Point P 1.9 — Although the terminology is used in the mouse phenotyping community, it is confusing to use “annotate” to mean “call a gene positive for a phenotype” - in the wider community “annotate” means to test a gene for a phenotype and to call the result either positive or negative (thereby distinguishing it from missing data). This specialised use of the word "annotation" should either be prominently flagged or replaced by e.g. “positive annotation”.

Reply: Good point...we are now using the terminology “calling [phenotypic] hits” which we introduce here:

> *The primary scientific goal is to identify statistically significant knockout-induced phenotypic perturbations, also referred to as phenotypic* hits *or* positive annotations. *(page 2, lines 52-53)*

Reviewer Point P 1.10 — It is unclear from the methods how centre effects are modelled. Are they fixed or random effects, or are they ignored at the UV stage and investigated later? Are they controlled for, eg in the selection of control animals?

Reply: Yes, centre effects are controlled for at the UV model stage where a stratified analysis (centre-by-centre) is performed. Also, each synthetic null line is generated only from mice at the centre corresponding to the matching KO line. These approaches to modelling centre effects are clarified in the text as follows:

1. To clarify that the UV model is fitted to data from one (centre, phenotype) combination of the time we say:

   We fit this model to each (phenotype, centre) combination separately, yielding an estimate (and SE) of the phenotypic perturbation, $\hat{\theta}^{\mathsf{UV}}_{pg}$ (and $\hat{s}^{\mathsf{UV}}_{pg}$), for each (phenotype $p = 1, \ldots, P$, gene $g = 1, \ldots, G$) pair at which measurements are available. (page 3, lines 104-107)

2. To clarify that the generation of synthetic null lines is performed with reference to centre we write:

   Synthetic null lines are generated by randomly selecting groups of WT animals from a single centre so as to match the experimental design characteristics of a particular true KO line at that centre. (page 16, lines 588-590)

Reviewer Point P 1.11 — Similarly where a gene is tested at multiple centres, are the data treated like different genes and then compared later on?

Reply: Yes, that's right, when a gene is tested at multiple centres the analysis treats the data as if they are from different genes and compares them later; we have clarified here:

> *We analyse them under the UV and MV models while ensuring the models are blind to their correspondence to one another as replicated samples. After analysis, we reveal the reference lines, and examine the replicability of findings on the same reference line across multiple phenotyping centres. (page 6, lines 230-233)*

Reviewer Point P 1.12 — Sex effects are removed at the UV stage and treated essentially as nuisance variables. But we think from earlier analyses of IMPC (ref 5 in the current MS) that about 12% of genes have sex-specific effects (ie an interaction), which is not handled by a simple sex effect that affects cases and controls equally, and which is what was presumably fitted in the MS. So far as I can see by the MV stage of the analysis it is impossible to see where sex has any effect, because the UV step removes these effects. At the very least, this requires some discussion.

Reply: Yes, we only include the additive effect of sex in our UV model. So, in cases of sex-specific effects, our UV estimator $\hat{\theta}^{\mathsf{UV}}_{pg}$ is targeting a weighted average of the male and female effects, with weights depending upon the relative sample size. We have added the following:

> *In cases where genotype effects differ between sexes [5], θ_{pg} is interpretable as the average of those sex-specific effects. (page 3, lines 136-137)*

In addressing your comment, we did analyse the data for sex-specific effects (under both UV and MV models) and found very few instances of significant genotype-sex interactions. Upon more detailed investigation, our interpretation is that some of the significant results in the Nature Communications sexual dimorphism paper (interpreted there as sex-specific effects) may have been rejecting the null because of a complex relationship between sex and phenotype *in wild-type animals alone*. In other words, WT animals appear to exhibit non-additive effects of sex on phenotype, and the null model used in the Nature Comms paper may not be flexible enough to capture the complex sex-phenotype interplay in WTs, leading to rejection of H_0 in favour of a more complex model, even if a KO gene does not impact a phenotype in a sex-specific way. Happy to go into more detail, please just reach out directly.

We propose that our current MV paper is not the right forum and doesn't have the scope to discuss this. We will discuss these new findings (questioning the existence of widespread sex-specific effects) with colleagues such as you who co-authored with us on the sexual dimorphism paper, and we can collectively investigate this idea that some of the sex-specific effects we have identified so far may be false positives.

Reviewer Point P 1.13 — A major analysis hurdle for the IMPC data is the correct selection of controls. This happens upstream of the other analyses in the current MS (I think) so it is impossible to tell if the selection method used in the current study is correct (but equally, no

reason to suppose it is problematic either). There has already been some work on this problem (ref 9 in the MS). It is not clear if the method of choosing controls in the current analysis differs from that done previously and whether the phenotype calling is affected. In the Methods in the UV analysis states that it is "...fitted only to data from KO line g accompanied by data from the entire rolling baseline of WT animals...". Does this mean all controls are compared to a given KO? But later the Methods describes Negative Control Data as "We generate negative control data and use them to enhance inference under the univariate (UV) and multivariate (MV) models. Negative-control lines are generated by randomly selecting groups of WT animals so as to match the experimental design characteristics of a particular true KO line..." This is confusing: please clarify what is going on. It appears in the Calibration section of the Methods as if negative controls were treated as if they were positives for calibration of thresholds. This is different from defining a set of controls for each gene. It is essential to describe and justify the control selection strategy used (see also comments about centre effects, above).

Reply: Yes, thanks for this point, which has motivated us to describe more carefully the use of WT animals throughout the analytical pipeline. The answer to your question "Does this mean all controls are compared to a given KO?" is: Yes, this comparison is performed – within each centre – in the UV analysis.

To address your comment, we have improved our presentation of how WT data are incorporated into the analysis. We now use the terminology "control animals" to refer to the WT animals measured contemporaneously in phenotyping centres. We no longer use the terminology "negative control lines" which, as you pointed out, is ambiguous. We define the specific terminology "synthetic null" line in Methods section 5.3.1 to refer to the artificial data we generate. We have introduced mathematical notation for the groups of randomly selected animals (WT and KO) that are incorporated at various stages of our cross-validation framework (Section 5.3).

Reviewer 2

Introduction

In this paper, the authors develop and apply a statistical framework for the joint analysis of high-dimensional phenotype response studies. Accurate statistical methods for the analysis of response data are very much needed, and the paper describes an application to a major data resource as part of the international mouse phenotyping consortium. The paper is well written and of broad interest.

The paper is a combination of a new method and an application, although as presented the method is at the center. My main concern is the methodological contribution and the conceptual novelty of what is presented. As correctly referenced and stated, there have been alternative frameworks proposed to essentially perform the same type of analysis. While previous methods have been applied to somewhat different questions and data, e.g. multi-tissue expression QTL mapping, the statistical principles are clearly transferrable and it is not clear how the presented method relates and compares (author reply in P 2.1 below).

Comments

Reviewer Point P 2.1 — Relationship to cited prior work. The approach proposed by Urbut et al. (2019) is quite similar to what is presented here, yet a comparison and even a discussion to relate the approach is missing. Given that this method could in principle also be applied to this study, a comparison would seem necessary. This may impact the relative weight of the methodology that is presented versus the application to the specific dataset, which however is not a problem per se.

Reply: Yes, thanks for your suggestion which has led to substantial improvement in our methodology and the paper. We have reoriented our methods to build clearly on existing work, not only the paper you suggest by Urbut *et al.* (2019) [20], but also work by Bovy *et al.* [21]. We compare and contrast our now-enhanced methods against these existing approaches. A qualitative comparison shows our methodological extensions to yield higher performance according to metrics such as better hit rates, replicability and cross-validated likelihoods (please see Methods–*Comparison with existing methods*)

Reviewer Point P 2.2 — Scope of the paper and the method. The title and abstract are framed very general, yet the application that is considered is specific. Depending on how the authors decide to address the previous comment, it may be useful to consider additional application to additional data to demonstrate broad applicability.

Reply: Thanks for this suggestion – we have now analysed the additional eQTL data set of Urbut *et al.* (2019) [20], performing a benchmarking comparison based on cross-validated likelihood (this follows Urbut *et al.*'s approach to checking model fit).[4] We present the results of the eQTL benchmarking alongside the other comparisons and benchmarking we perform in Methods–*Comparison with existing methods.*

Reviewer Point P 2.3 — Downstream analysis of Sigma. The factor analysis downstream modelling is interesting, as is the comparison of direct phenotypic correlation versus the estimated effected size covariance structures. These analyses are based on potentially noisy ML-based estimates of Sigma, and it is not clear to me if and how the uncertainty in these estimates is accounted for in these analyses. At the very least some sort of sensitivity analysis should be conducted to demonstrate robustness.

Reply: Yes, good point, we have now included Methods–*Sensitivity analysis.* This is now one of several new model checks and sensitivity analyses in the revised text.

[4] We did look into comparing sensitivity to detect eQTLs (analogously to our hit rate benchmarking) but we could not identify a way of controlling error rates appropriately, e.g. it's not clear to us on how to create a nonparametric null distribution in this context. There could be a nice way of doing this based on the raw data (as opposed to the estimated eQTL effect-size data taken as input to mash and our MV model), but this is beyond the scope of our current paper.

Reviewer 3

Introduction

The authors tackle the problem of inferring an annotation map from genes to multiple phenotypes at a genome-wide level using knockout mouse lines. Uniquely, they propose a two-step procedure to model all phenotypes jointly while also imputing missing perturbations, and they demonstrate the applicability and merits of their approach in an extensive case study. The work is well motivated in terms of the growing need to characterise and leverage the dependence structures across multiple phenotypes in order to both enhance statistical power and provide a holistic description of the regulatory mechanisms shared across traits.

I feel that this paper has the potential to make an important contribution to the field if the benefits of the MV modelling approach – and the assumptions on which it relies – are more carefully assessed in the context of the presented study. In particular, while the authors rightfully stress that joint modelling will be beneficial in presence of shared regulatory patterns across phenotypes, they do not discuss the fact that, under their MV model, the borrowing of information is a direct consequence of assuming a shared perturbation covariance structure for all KO lines. A thorough discussion on the appropriateness of this key assumption for the presented study is lacking at present. Likewise, the very high rate of missingness (55%!) in the data raises questions about the reliability of the multivariate analysis and the imputation process. These two unanswered questions leave the reader with a sense of overinterpretation of the merits of the MV approach. The authors should provide additional checks and discussion, and they should formulate words of caution where firm conclusions cannot be reached. In more details, the following points should be addressed:

Major comments

Reviewer Point P 3.1 — The model assumes that the covariance structure $\boldsymbol{\Sigma}$ of the phenotypic perturbations $\boldsymbol{\theta}_{\cdot g}g$ is the same for all KO lines g. This is a very strong assumption, whose appropriateness in the context of the presented real data should be evaluated. To bring insights as of whether this is reasonable, a careful empirical comparison, based on the UV summary statistics, of the phenotypic correlation matrices across different random subsets G of knockouts should be added (subsets of size 500, say, should provide reliable estimates). For instance, the authors should assess the variability of the estimates $\left\{\hat{\Sigma}_{\mathcal{G}}\right\}$ using the Mahalanobis distance or other metrics, and then compare the MV results obtained using the original pulled estimate $\hat{\Sigma}$ and one estimate $\hat{\Sigma}_{\mathcal{G}}$ particularly far from it and they should check whether the final conclusions differ or not.

Reply: Thanks for the great suggestion, which we have now implemented. Details are written up in Methods–*Data subsampling*. In brief, we find the downstream results to be qualitatively similar between the two most KL-divergent sub-data sets of size 500.

Reviewer Point P 3.2 — The description of the imputation approach and the assessment of its accuracy are unconvincing: the authors should clearly state the type of missingness assumed (MCAR, MAR, MNAR) and discuss why it is appropriate in the context of the presented data.

Reply: Your useful input has led us to better clarity on how we are treating missing data. We have replaced the terminology "imputation" throughout the manuscript with various paraphrases of "inference in the presence missing data", the latter being a better reflection of what we're doing. In Methods–*Missing-data caveats/guidelines*, we have now incorporated text on: the assumption of MAR; why it is intuitively appropriate for the bulk of our data; and how we perform (indirect) checks that the inference is performing well when data are missing:

> *Estimation of $\boldsymbol{\theta}_{\cdot g}$ when $\hat{\boldsymbol{\theta}}^{UV}_{\cdot g}$ is only partially observed can be performed coherently provided the statistical model is well specified with respect to the underlying data generating mechanism, and provided the unobserved data are missing at random (MAR) [43, 44, 21]. While there is a large proportion of missing data, it is clear from Figure 2 that the bulk*

of data is missing in obvious blocks and is a result of certain measurements/procedures not being performed in some centres. In this context of certain centres systematically not performing a subset of measurements, the MAR assumption is reasonable, in that the missing data mechanism, "given the missing data and the value of the observed data, is the same for all possible values of the missing data." [43]. In spite of this reassuring observation, there is naturally still going to be some relatively small proportion of data that violate the MAR assumption in such a large and complex data set as this. We therefore recommend additional checks on how practically reasonable the MAR assumption is. These are described in Results–Validating replicability (with reference to Figure 6(c-d)), and in Methods–Predicting masked data. Our recommendation to practitioners is carefully to examine the appropriateness of the MAR assumption in their particular context in the light of the work of Rubin and colleagues [43, 44]. If there are any doubts about the MAR assumption's validity, we recommend further empirical checks; particular, the cross-validated mask and predict approach described in Methods–Predicting masked data can be implemented in a wide variety of multivariate datasets with missing data, and we recommend this as a tool for checking accuracy post-hoc when the rate of missingness is high. (page 13, lines 497-515)

Reviewer Point P 3.3 — Figure 4c shows that the sensitivity obtained after imputation is lower to that of the UV method for certain procedures. This is worrisome and should question the appropriateness of imputing for the presented data, especially given the percentage of missingness of 55%. The authors should explain why imputation would still be appropriate here or they should explicitly mention that it isn't, at least for those phenotypes where the sensitivity is low. Some discussion is already given on page 5 but it should be strengthened. In Supplementary Figure 4, please display the percentage of missing KO lines for each phenotype. Please also review all the other plots and stratify them by the level of missing data, where appropriate.

Reply: We like your recommended idea of displaying the percentage of missing KO lines which we have now done in Supplementary Figure 4. Please note that the results in Figure 5(c) have changed in the revised manuscript because of different methods implementation (i.e. the new more sophisticated multiscale covariance mixture model inspired by [20]); also please note we are now plotting the "MV model (imputed)" and we have clarified in the legend that the imputed and UV model are nonoverlapping (and hence that it is not contradictory to have imputed straying below the UV hit level, which it does on occasion).

Reviewer Point P 3.4 — Linked to the previous comment: the authors should provide clear caveats/guidelines as of (1) when imputation is deemed inappropriate and (2) how to assess its accuracy post hoc. This information is crucial to prevent overinterpretation, especially when the shared structure across phenotypes/perturbations is weak and when the rate of missingness is high.

Reply: Yes, good point, we have now included caveats/guidelines and suggestions for assessing accuracy post hoc in Section 5.1.1.

Reviewer Point P 3.5 — The replication of the results in independent studies as presented in Figure 5 is unpersuasive. Many of the annotations whose MV estimates are found to be consistent across studies correspond to imputed estimates. This agreement is thus likely to be the result of an imputation artefact arising from the underlying assumption of the shared phenotypic dependence of perturbations across knockouts. The authors should acknowledge this clearly and provide the cross-study replication rates separately for the imputed and non-imputed estimates.

Reply: Thanks, showing the cross study replication stratified by whether inference is performed on missing or observed data it is really important, and we have now updated Figure 6 and its legend to make readers more aware of this important aspect of the replicability results. The picture that the figure presents as a reassuring one – We see that false-sign rate estimates ($\widehat{\text{Fsr}}_{\text{replicate}}$) are low across all strata and compatible with intended level of error rate control.

Reviewer Point P 3.6 — I understand from the Section Hypothesis testing that the same procedure was employed to estimate the FDRs for the UV and MV model outputs, which in

principle should rule out the risk for the UV FDR estimates to be more conservative than the MV ones (say). However the MV assumption of a shared phenotypic correlation of perturbations across knockouts may increase the number of annotations and reduce the uncertainty of estimates, thus leading to inflated detection rates at a given FDR. Given that real data are used – where the ground truth is unknown – it is not clear that the new annotations detected are genuine, and not solely a consequence of the assumption on the shared phenotypic structure. Please also address the next point to clarify this and better demonstrate that the proposed method is statistically more powerful.

Reply: Thanks, this really helpful comment has focused our minds on presenting a convincing, coherent account of why we think these MV hit calls are truly exhibiting high power under a well-controlled error rate. These changes in revision have helps clarify and strengthen this case:

1. The replicability comparison across pairs of replicates with one centre having missing data vs. the other centre having measured data, shown in Figure 6(d), suggests a reasonable control of the false-sign rate under the circumstances of concern that you identify.
2. The data subsampling checks in Section 5.4.2 demonstrate that results are stable and do not show discordance across training sub-datasets of size 500 (compared to our full training data set of size 2000). This provides some evidence that the new annotations are genuine.
3. The MV model in the revised manuscript at (2)-(3), now being a multi-scale mixture of covariances, relaxes the assumption of a shared covariance structure across all knockouts.
4. We see a consistent, replicable, biologically reasonable story emerging (Sections 3.8, 3.6, 3.7).

Reviewer Point P 3.7 — The discussion provides a series of numbers on the general performance of the method, e.g., sensitivity rates and annotations detected (in particular on pages 4-5). This is good but I am missing some biological examples/interpretation of the results. As the paper presents a case study, the authors should provide sound examples that illustrate why the MV method brings more biological insights/findings compared to a classical UV method. In particular, when the appropriateness of certain assumptions is difficult to assess statistically, highlighting biologically sensible results will provide an additional level of support for validity of the MV approach.

Reply: Great suggestion, thanks, we have now added various sections to address this. In Results–*Gene Ontology co-enrichment* we find a far greater enrichment of GO terms with the MV model vs the UV model, Also, the instances of co-enrichment between the GO-terms and the IMPC phenotypes seem biologically sensible.

Minor comments

Reviewer Point P 3.8 — On page 4, the authors mention that the UV and MV models annotate most perturbations with the same directionality, yet they disagree for 45 of the cases. It would be interesting to investigate further what happens for (a subset of) these 45 cases. What in the MV model would drive these differences? For the cases considered, which directionalities would be biologically more sensible?

Reply: Under the new refined model, we observe no differences in hit calling between the UV and MV models. We do see 3 disagreements between the MV model and the existing IMPC database, as described in Section 3.6, and there we investigate these according to your suggested approach. Unsurprisingly for such a small number, there is not any persuasive evidence either way in support of the MV or IMPC database outputs being more biologically sensible from a directional perspective.

Reviewer Point P 3.9 — Any attempt to merge the two modelling stages (i.e., a unique joint model applied directly to the raw data rather than to the UV statistics)? Clearly, this has the potential to further enhance statistical power as it would avoid the loss of information resulting from the first univariate step. The authors mention that such an approach would be computationally prohibitive but it would be good to point to the literature on earlier attempts that suggests so.

Recent work indicates that devising a clever optimisation strategy might yield scalable inference, in particular when using expectation-maximisation as done by Deshpande et al. (2019) in the context of sparse multivariate regression.
Please provide more discussion, cite previous attempts and, if possible, compare the method to these on a subset of the data.

Reply: We now build on existing two-stage methodologies XD and mash [21, 20] which places our work firmly in that vein of research. Also, to discuss the potential for joint modelling in this context we have added this text:

> *An even more ambitious goal would be to fit a full multi-level factor model directly to the raw data [37, 38, 39, 40, 41], i.e. to target the posterior* $p(\boldsymbol{\Sigma}, \boldsymbol{R}, \boldsymbol{\Theta} \mid \boldsymbol{Y})$*, where* $\boldsymbol{Y}$ *is the raw, animal-level data. This is in principle extremely attractive, as it would potentially allow for more information to flow the raw data to the parameters of interest, and could deliver more power. Of course, effective inference would rely upon the (more complex) model being a sufficiently good representation of the data. With reference to the multilevel UV model at (1), a joint multi-level factor model would probably require an inter-measurement* $(P \times P)$ *covariance structure underlying each of the different random effects; this would be nontrivial to implement, especially with non-identifiability considerations. A more basic challenge is the size of the data set increasing by an order of magnitude, which could have a considerable impact on the computational complexity, depending upon the implementation. While the scope of this paper is to build on and extend the modular framework of [21, 20], we do see joint multilevel factor modelling as a promising area to explore in future, especially with the ongoing development of scalable optimisation methods for complex models [42]. (page 13, lines 461-474)*

Reviewer Point P 3.10 —

1. Any evidence from the posterior output that the simultaneous estimation of $\boldsymbol{\Sigma}$ and $\boldsymbol{R}$ is subject to identifiability issues (especially given the uninformative prior distributions used for both)?

2. Have the authors considered using structured covariance matrices (some regularisation may be useful if the number of phenotypes is large)? On page 8, they mention: *We require a sufficient number of independent multivariate observations (in our case KO lines) to estimate the covariance structure in* $\boldsymbol{\Sigma}$ *and* $\boldsymbol{R}$ *effectively. If there are insufficient data to estimate full* $P \times P$ *covariance matrices, then* $\boldsymbol{\Sigma}$ *and* $\boldsymbol{R}$ *could be represented more parsimoniously using reduced-rank factor models.* Have the authors tried this, and would inference be sensitive to such structural choices?

3. Also: have the authors considered estimating R from the data upfront? For instance by constructing an empirical correlation matrix from a subset of UV estimates that may be attributed to the null? Are the authors referring to a similar idea when writing (in the *Negative control data* Section): "The negative control lines in $\mathcal{G}_1^-$ are used for inference in the MV Bayesian model (3) under the assumption that they truly had no genetic perturbation, i.e., $g \in \mathcal{G}_1^- \Rightarrow \boldsymbol{\theta}_{\cdot g} \equiv \mathbf{0}$, thereby aiding estimation of $\boldsymbol{R}$." Please provide more detail.

Reply: Thanks for another really astute comment, and we have now implemented these suggestions, specifically we are now working with a reduced rank factor representation, and we are estimating $\boldsymbol{R}$ upfront. Answering your points in turn:

1. We did observe some partial non-identifiability in the previous version of methods but this is now moot as we are now fixing $\boldsymbol{R}$ upfront, as per your suggestion and noting also that this approach was taken by the creators of `mash` [20]

2. Yes, we have now implemented a multi-scale mixture of reduced-rank factor $\boldsymbol{\sigma}$, With details given in Methods–*Multivariate model*, particularly around equation (4).

3. Yes, we have now implemented this following your suggestion and noting [20] adopt a similar approach.

Reviewer Point P 3.11 — EM algorithm: in the Supplementary Material the authors mention that they average across multiple instances of an EM run for 500 iterations to alleviate the problem of runs trapped in local modes, and that the values of the converged objective function are "similar" anyway across these instances. Why 500 iterations? To have a better control on the convergence status, it would be better to use a stopping criterion based on a tolerance on the changes of the objective function value.

Reply: Thanks for this good suggestion, which we have implemented:

> *The EM algorithm is deemed to have converged when the change in objective function between consecutive iterations falls below a tolerance threshold. We choose the tolerance threshold adaptively, with reference to variation in log likelihood contribution across samples. Specifically, denoting the contribution of the gth sample to the log likelihood at the tth iteration by $l_g^{(t)}$, the tolerance is set to $tol := \varepsilon_{tol} N_{tra} MAD(\{l_1^{(t)}, \ldots, l_{N_{tra}}^{(t)}\})$, where $MAD()$ denotes the median absolute deviation, N_{tra} the number of training samples, and ε_{tol} a user-specified constant (we used $\varepsilon_{tol} = 10^{-4}$). (page 15, lines 559-564)*

Reviewer Point P 3.12 — It would be interesting to assess whether the estimates of $\boldsymbol{\Sigma}$, $\boldsymbol{R}$ and $\boldsymbol{\theta}$ obtained by the five different runs agree or disagree to the point that they would lead to different conclusions. It is not clear to me whether the proposed initialisation procedure provides starting points that are sufficiently representative of the posterior landscape. What happens if this procedure is replaced by a vanilla random initialisation for all parameters? Have the authors performed any sensitivity analysis suggesting that five parallel runs is sufficient?

Reply: You'll see that we have enhanced the entire inferential framework in response to all the really helpful reviewer comments. Within this new cross-validated framework, we run a single EM optimisation for each fold. We perform sub- sampling cross checks in Section 5.4.2 which check for sensitivity not only to differences in initialisation but also to differences across sub-datasets. We like your suggestion above of investigating random initialisation. When we implemented we see that supervised initialisation it is indeed helpful for convergence to "better" optima (better in terms of higher cross-validated likelihood) and we make this point in Section 5.2.1 by comparing CV likelihoods between supervised initialisation and vanilla random initialisation on the same dataset, finding supervised initialisation to outperform random.

Reviewer Point P 3.13 — Finally, in order for the contribution to be more impactful and the work reproducible, the authors should provide a software for their method, e.g., on GitHub.

Reply: Data and software to reproduce the analyses performed in the paper are available at `https://github.com/georgenicholson/multivariate_phenotype_data_and_code`.

Conclusion

In conclusion, the paper makes an important contribution to gene knockout analysis for multiple phenotypes, and illustrates the benefits of joint modelling in a concrete real-world study. To convince on the immediate usefulness of the method, the authors should better assess (1) the validity and impact of key modelling/missingness assumptions, (2) the performance improvement over UV methods in light of these assumptions and (3) the replicability of the results, via additional numerical experiments and a discussion of the biological relevance of the findings.
