# Supplementary material for: Multivariate phenotype analysis enables genome-wide inference of mammalian gene function
Source: PLoS Biol. 2022 Aug 9;20(8):e3001723. doi: 10.1371/journal.pbio.3001723 (PMC9391051; doi:10.1371/journal.pbio.3001723)
Supplement: Attachment — Submitted filename: reviewer_response_revision_2.pdf [file pbio.3001723.s015.pdf]

Reviewer 1

Oxford, 16th June 2022

Dear Richard,

Thank you so much for kindly dedicating your time and effort to reviewing and much improving our paper—we really appreciate it!

We do hope that we have addressed all your comments satisfactorily and that you are now happy with the manuscript.

A further point in addition to our responses to your comments below: may we please draw your attention to a small correction we have made in the UV model description in this second revision (lines 139 and 1085 in tracked_changes_revision_2.pdf)? In the first revision, we forgot to update the UV model description to indicate that we are now adjusting for sex-genotype interaction in the UV linear model, a change we made in response to your first round of comments. We don't want to focus this paper on the sex-genotype interaction effects themselves (for reasons we described in response to Reviewer Point P1.12 in your first set of comments) but adjusting for them is the approach we have now taken, and of course we should make that clear in the main Results section and in Supplementary Note 1.

With very best wishes,

George Nicholson (on behalf of all co-authors)

Comments on revised version

Reviewer Point P 1.1 — The Methods contains some important results on robustness of the results, in particular the cross-validations to show that missing data are imputed satisfactorily. I suggest you move at least a summary of these analyses into the main Results, since you raise the problem of missing data in the introduction but don't directly address it in the main Results in the current revision. Line 230 near Fig 2 might be a good place to introduce this.

Reply: Thanks for this nice idea, which we have implemented exactly as you suggest. We have now moved the bulk of the missing-data discussion from Methods to join the missing data results in a new main Results section called *Inference when data are missing*, leaving only the technical details of the missing-data MV model in a Methods section called *MV model when data are missing*.

Reviewer Point P 1.2 — Explain clearly in the Results what the categorisation of calls as -1, 0, 1 represents (eg legend to Table 1) - this seems to be buried in the Methods

Reply: Great spot, thanks, we have now added the following clarification to the legends of Tables 1 and 8:

> *We represent calls by a number in* $\{-1, 0, 1\}$*, with* 1 *and* -1 *denoting significant positive and negative phenotypic perturbations respectively, and* 0 *denoting a lack of statistical significance.*

Reviewer Point P 1.3 — Explain more clearly what the factor analysis means from a biological perspective. I took it to mean there are clusters of phenotypes that tend to get called in the same way (is that correct?). In the abstract they are described as "sparse factors" which has an obscure technical meaning, losing clarity.

Reply: Yes, your interpretation's correct. We have improved the clarity in the abstract, and removed the word "sparse", writing:

A factor analysis of the MV model's fitted covariance structure identifies 20 clusters of phenotypes, with each cluster tending to be perturbed collectively. These factors cumulatively explain 75% of the KO-induced variation in the data, and facilitate biological interpretation of perturbations.

We have also improved the presentation in Section 3.10:

An eigendecomposition of the MV model's fitted covariance structure ($\hat{\Sigma}_{pooled}$ of (26) in Methods–Cross-validation and model averaging) shows that 75% of the correlation structure is explained by the first 20 eigenvectors; Supplementary Figure 8 plots the cumulative variance explained. We rotate these eigenvectors to a sparse, interpretable set of loadings, or factors, which are visualised in Figure 8(a). The important notion of sparsity in this context, illustrated in Figure 8(a), is that the vast majority of phenotypes at any particular factor have loadings close to zero (i.e. they are coloured green). Each factor therefore defines a small cluster of phenotypes that have large positive or small negative loadings. From a biological perspective, each cluster of phenotypes tends to be perturbed collectively by gene KOs.

By examining each cluster of phenotypes, and taking into account the signs of its loadings, we manually curate labels describing the biological interpretation of each factor. For example, the first factor defines a cluster according to negative loadings on Bone Mineral Content, Bone Area, Lean mass, Body length, and Heart weight; this factor is therefore labelled 'Body size (-)'. Factor labels are shown on the axes of Figure 8(b). The suffix (+) or (-) denotes the directionality of effect implied by the sign of the loadings in Figure 8(a), e.g. 'Body size (-)' denotes 'Reduced Body size' and 'Deafness (+)' denotes 'Increased deafness'.

Reviewer Point P 1.4 — In Fig 1, it might be helpful to show both the UV and MV estimators and their confidence bands, for illustrative purposes. In general, are the MV estimates more precise than the UV? (sensu smaller standard errors).

Reply: Thanks for the helpful suggestion; we have now added UV and MV estimators and their confidence bands and updated the legend to describe the new features and also to answer your question on relative precision: yes, the MV are almost always more precise, while also being usually shrunken towards zero:

Experimental design of the IMPC. Each point corresponds to one animal, with data from two KO lines—labelled g and $\tilde{g}$—displayed alongside contemporaneous data from a large number of control (wild-type, or WT) animals in grey (see legend). Panels (a) and (b) show data from phenotypes p (Triglycerides) and $\tilde{p}$ (Body fat percentage) respectively. Our goal is to quantify the underlying expected perturbations of the red/blue coloured points from the rolling WT baseline (illustrated with a smooth black curve), in the presence of structured experimental noise. Annotated on the plot, to the right of the red/blue measurement data of each gene-phenotype pair, are the posterior mean estimates from the UV and MV models, $\hat{\theta}_{pg}^{UV}$ with empty squares and $\hat{\theta}_{pg}^{MV}$ with filled squares (see legend), along with error bars denoting ± 2 posterior SDs. In the current paper we combine the information in $\hat{\theta}_{pg}^{UV}$ across multiple related phenotypes, such as p and $\tilde{p}$, thereby generating improved estimators $\hat{\theta}_{pg}^{MV}$. The relative means and SDs of the UV and MV estimators shown in the plot are illustrative of their general properties—MV posterior means are shrunken towards zero (towards the black curve here) relative to the UV posterior means in 90.2% of cases (phenotype-gene pairs); while MV posterior SDs are smaller than UV posterior SDs in $> 99.9\%$ of cases.

Reviewer Point P 1.5 — In the abstract there is a mention of "7.5-fold increase in statistical power". It's not clear that this really means "There are 4,256 (1.4% of 302,997 observed-data measurements) hits called by the UV model, compared to 31,843 (10.5%) hits in the observed-data results of the MV model". Suggest you expand the text in the abstract.

Reply: Thanks, great suggestion, we have changed the abstract as you suggest.
